# Effects of Fe_3_O_4_ Magnetic Nanoparticles on A549 Cells

**DOI:** 10.3390/ijms140815546

**Published:** 2013-07-25

**Authors:** Masatoshi Watanabe, Misao Yoneda, Ayaka Morohashi, Yasuki Hori, Daiki Okamoto, Akiko Sato, Daisuke Kurioka, Tadashi Nittami, Yoshifumi Hirokawa, Taizo Shiraishi, Kazuaki Kawai, Hiroshi Kasai, Yukari Totsuka

**Affiliations:** 1 Laboratory for Medical Engineering, Division of Materials and Chemical Engineering, Graduate School of Engineering, Yokohama National University, Yokohama 240-8501, Japan; E-Mails: qqqmoro@gmail.com (A.M.); c17cssbos@gmail.com (Y.H.); dokamoto615@yahoo.co.jp (D.O.); akko.613@gmail.com (A.S.); kuri321@gmail.com (D.K.); nittami@ynu.ac.jp (T.N.); 2 Department of Pathologic Oncology, Institute of Molecular and Experimental Medicine, Mie University Graduate School of Medicine, Tsu 514-8507, Japan; E-Mails: xyxfp209@yahoo.co.jp (M.Y.); ultray2k@clin.medic.mie-u.ac.jp (Y.H.); tao@doc.medic.mie-u.ac.jp (T.S.); 3 Department of Environmental Oncology, Institute of Individual Ecological Sciences, University of Occupational and Environmental Health, Kitakyushu 807-8555, Japan; E-Mails: kkawai@med.uoeh-u.ac.jp (K.K.); h-kasai@med.uoeh-u.ac.jp (H.K.); 4 Division of Cancer Development System, National Cancer Center Research Institute, Tokyo 104-0045, Japan; E-Mail: ytotsuka@gan2.res.ncc.go.jp

**Keywords:** magnetic nanoparticles, cytotoxicity, genotoxicity, A549, CD44

## Abstract

Fe_3_O_4_ magnetic nanoparticles (MgNPs-Fe_3_O_4_) are widely used in medical applications, including magnetic resonance imaging, drug delivery, and in hyperthermia. However, the same properties that aid their utility in the clinic may potentially induce toxicity. Therefore, the purpose of this study was to investigate the cytotoxicity and genotoxicity of MgNPs-Fe_3_O_4_ in A549 human lung epithelial cells. MgNPs-Fe_3_O_4_ caused cell membrane damage, as assessed by the release of lactate dehydrogenase (LDH), only at a high concentration (100 μg/mL); a lower concentration (10 μg/mL) increased the production of reactive oxygen species, increased oxidative damage to DNA, and decreased the level of reduced glutathione. MgNPs-Fe_3_O_4_ caused a dose-dependent increase in the CD44^+^ fraction of A549 cells. MgNPs-Fe_3_O_4_ induced the expression of heme oxygenase-1 at a concentration of 1 μg/mL, and in a dose-dependent manner. Despite these effects, MgNPs-Fe_3_O_4_ had minimal effect on cell viability and elicited only a small increase in the number of cells undergoing apoptosis. Together, these data suggest that MgNPs-Fe_3_O_4_ exert little or no cytotoxicity until a high exposure level (100 μg/mL) is reached. This dissociation between elevated indices of cell damage and a small effect on cell viability warrants further study.

## 1. Introduction

Nanotechnology—the manipulation and production of matter sized between 1 and 100 nm—has grown markedly with the promise of substantial benefits and applicability to such diverse areas as clothing, electronics, engineering, and healthcare [1]. The principal goal of nanotechnology is to develop new materials in the nanometer scale, including nanoparticles, defined as particulate materials with at least one dimension of less than 100 nm. The design and development of nanomaterials have been of fundamental importance to the industry, given their novelty and the benefits conferred by their physicochemical properties.

Magnetic nanoparticles (MgNPs) are a subclass of nanomaterials. Among MgNPs, Fe_3_O_4_-containing MgNPs (MgNP-Fe_3_O_4_; magnetite) are the only MgNPs approved for clinical use. Magnetite has a cubic inverse spinel structure with oxygen forming a face-centered cubic (FCC) closed packing; the interstitial tetrahedral and octahedral sites are occupied by Fe cations [2]. Due to their unique physical, chemical, and mechanical features, MgNPs-Fe_3_O_4_ have been used as magnetic resonance imaging contrast agents, targeted drug delivery systems, and hyperthermic agents when placed in an external magnetic field [3,4]. Modified/unmodified MgNP-Fe_3_O_4_ has been reported to improve the efficiency of anticancer drugs and reverse multidrug resistance [5,6]. However, these same properties of MgNPs can induce cytotoxicity and genotoxicity [7]. Studies have shown that MgNPs-Fe_3_O_4_ are less toxic than MgNPs containing SiO_2_, TiO_2_, CuO, and TiO_2_ [7–9]. However, results regarding the potential of MgNPs-Fe_3_O_4_ to induce cytotoxicity, genotoxicity, and oxidative stress, have been inconsistent [7–11], necessitating further study to identify any potential toxicity associated with their use. Therefore, the objective of this study was to investigate the cytotoxicity and genotoxicity of MgNPs-Fe_3_O_4_. Experiments were designed to examine the effect of MgNPs-Fe_3_O_4_ on indices of oxidative stress, and resultant cellular and nuclear damage in A549, human alveolar epithelial-like type-II cells. We also assessed the effect of MgNPs-Fe_3_O_4_ on the expression of CD44, a transmembrane glycoprotein involved in inflammation, cell migration, signaling, and tumor metastasis [12,13].

## 2. Results and Discussion

Studies regarding the toxicological impact of MgNPs-Fe_3_O_4_ have yielded disparate results, depending on the cell type, surface modification, cell medium composition, protein-MgNP interaction, and oxidation state of iron [7,14]. We evaluated the cytotoxic effects of MgNPs-Fe_3_O_4_ in A549 cells. We report that MgNPs-Fe_3_O_4_ caused LDH leakage only at a concentration of 100 μg/mL; increased ROS production and 8-OH-dG content, and decreased glutathione (GSH) levels were found with 10 μg/mL MgNPs-Fe_3_O_4_. Despite these responses, MgNPs-Fe_3_O_4_ caused only a small decrease and increase in cell viability and apoptosis, respectively.

### 2.1. Characterization of MgNPs-Fe_3_O_4_ Suspension in Various Conditioned Medium

**Figure 1:**
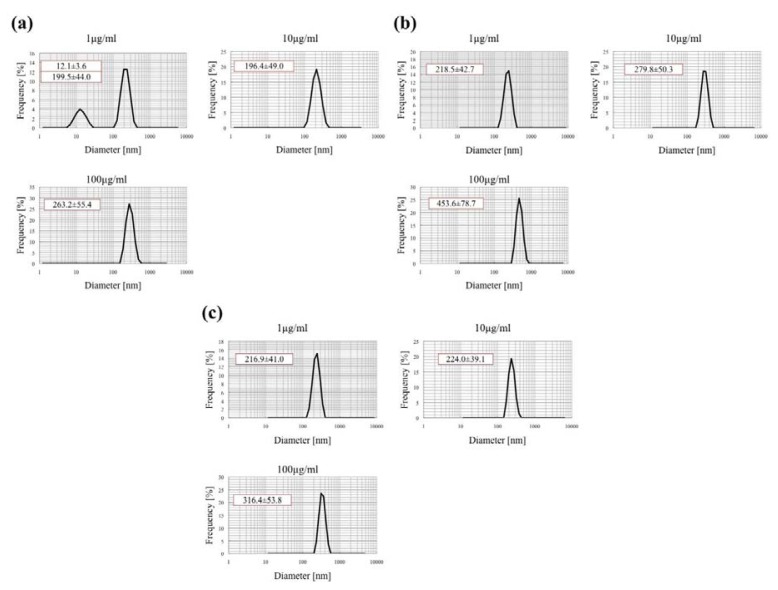
Measurement of MgNPs-Fe_3_O_4_ size by dynamic light scattering. MgNPs-Fe_3_O_4_ were suspended at a concentration of 1, 10 or 100 μg/mL in (**a**) Ham’s F-12 Medium with 10% fetal bovine serum (FBS); (**b**) Ham’s F-12 Medium alone; (**c**) Phosphate-buffered saline (PBS).

One of the most important significant factors for analysis of the toxicity of nanoparticles is size. As well as the sizes of the primary nanoparticles, the hydrodynamic sizes of secondary nanoparticles in dispersion are important as their sizes have a dramatic effect on cell response to exposure. The high ionic nature of the solution and the electrostatic/van der Waals interaction between protein and nanoparticles results in the formation of secondary particles. The mean hydrodynamic diameter of MgNPs-Fe_3_O_4_ in Ham’s F-12 medium, without FBS or supplements, increased in a dose-dependent manner (Figure 1b). In Ham’s F-12 medium with 10% FBS and supplements or PBS, the mean hydrodynamic diameter was comparable between MgNP-Fe_3_O_4_ suspensions of 1 and 10 μg/mL (Figure 1a,c); mean hydrodynamic diameter of MgNPs-Fe_3_O_4_ was greater at 100 μg/mL in both media. Together, these data suggest that MgNPs-Fe_3_O_4_ agglomerate at a high concentration. The presence of FBS appeared to enhance the stability of MgNPs-Fe_3_O_4_ in suspension. These data are consistent with a previous report showing that MgNPs show increased stability against aggregation in the RPMI-1640 with an increasing amount of FBS [15]. Therefore, the influence by the sedimentation rate of the secondary nanoparticles (NPs) and rations of protein to NPs could be taken into consideration in the *in vitro* toxicity of NPs. These results shows the hydrodynamic sizes of secondary nanoparticles in Ham’s F-12 medium with 10% FBS used in this study.

### 2.2. MgNPs-Fe_3_O_4_ Uptake

**Figure 2:**
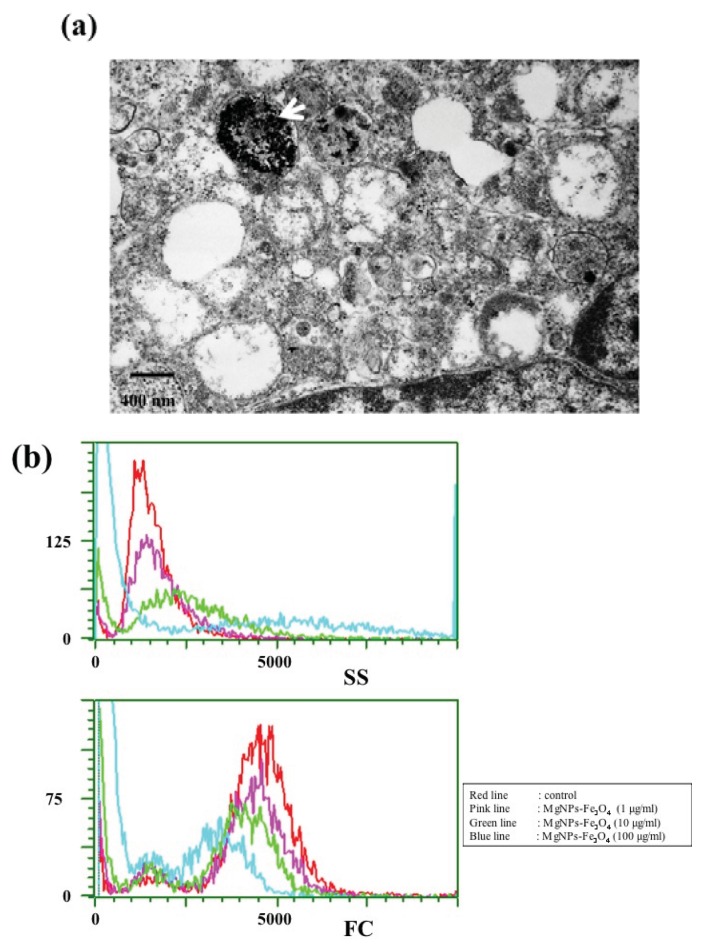
MgNPs-Fe_3_O_4_ uptake in A549 cells; (**a**) Transmission electron microscopy imaging of A549 cells treated with 10 μg/mL Fe_3_O_4_ magnetic nanoparticles (MgNPs-Fe_3_O_4_) for 24 h. MgNPs-Fe_3_O_4_ are enclosed in vesicles (arrow); (**b**) Analysis of MgNPs-Fe_3_O_4_ uptake by flow cytometric light scatter. A549 cells were treated with 0 (control), 1, 10 or 100 μg/mL MgNPs-Fe_3_O_4_ for 24 h. SS: side-scattered; FS: forward-scattered.

A representative micrograph shows that after 24 h, MgNPs-Fe_3_O_4_ aggregate within intracellular vesicles in A549 cells (Figure 2a). Figure 2b shows the flow cytometric light scatter histograms of the cells treated with the 0, 1, 10, or 100 μg/mL MgNPs-Fe_3_O_4_. The forward-scattered (FS) intensity (reflective of cell size) did not change; conversely, side-scattered (SS) intensity (reflective cellular uptake) increased in a dose-dependent manner. That is, the cells, which took up higher doses of MgNPs showed higher intensities of SS.

### 2.3. Effect of MgNPs-Fe_3_O_4_ on Cell Viability, Cell Membrane Damage, and Apoptosis

**Figure 3:**
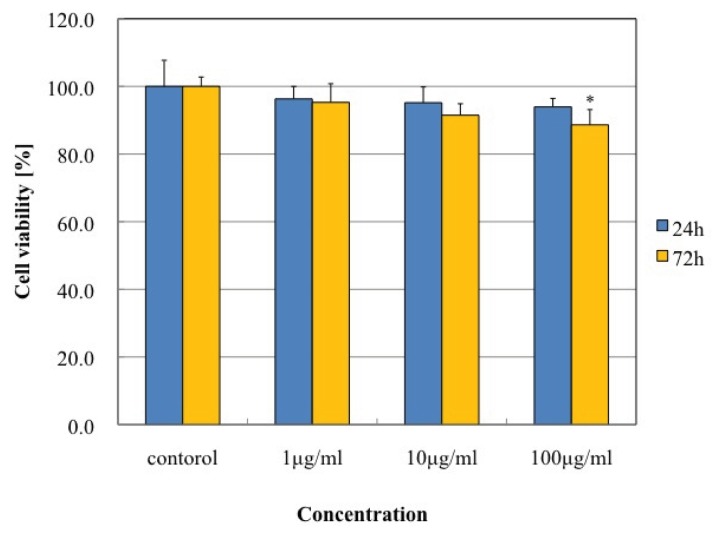
Effect of Fe_3_O_4_ magnetic nanoparticles (MgNPs-Fe_3_O_4_) on viability of A549 cells. A549 cells were treated with 0 (control), 1, 10 or 100 μg/mL MgNPs-Fe_3_O_4_ for 24 or 72 h. Cell viability was assessed using the Alamar Blue assay. Data are presented as the mean ± SD of 3 independent experiments. * *p* < 0.05 *vs.* control.

**Figure 4:**
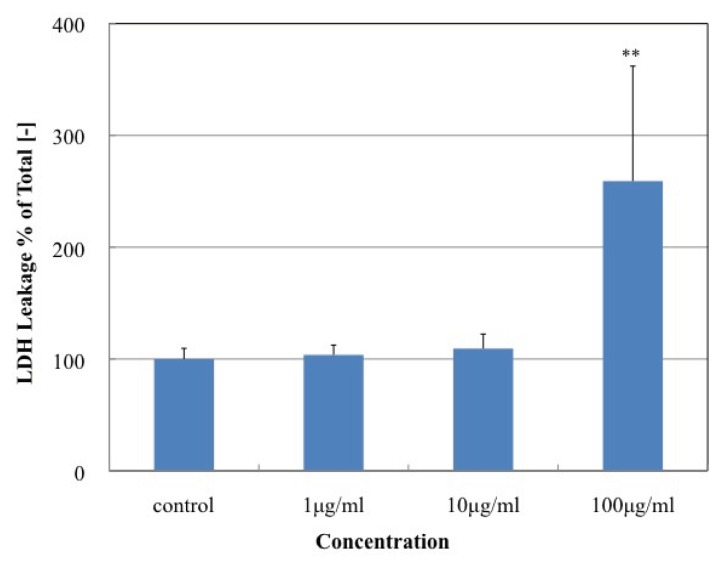
Effect of MgNPs-Fe_3_O_4_ on lactate dehydrogenase (LDH) release by A549 cells. A549 cells were treated with 0 (control), 1, 10 or 100 μg/mL MgNPs-Fe_3_O_4_ for 24 h. LDH release was assessed by formazan absorbance (LDH Cytotoxicity Assay Kit). Data are presented as the mean ± SD of 3 independent experiments. ** *p* < 0.01 *vs.* control.

**Figure 5:**
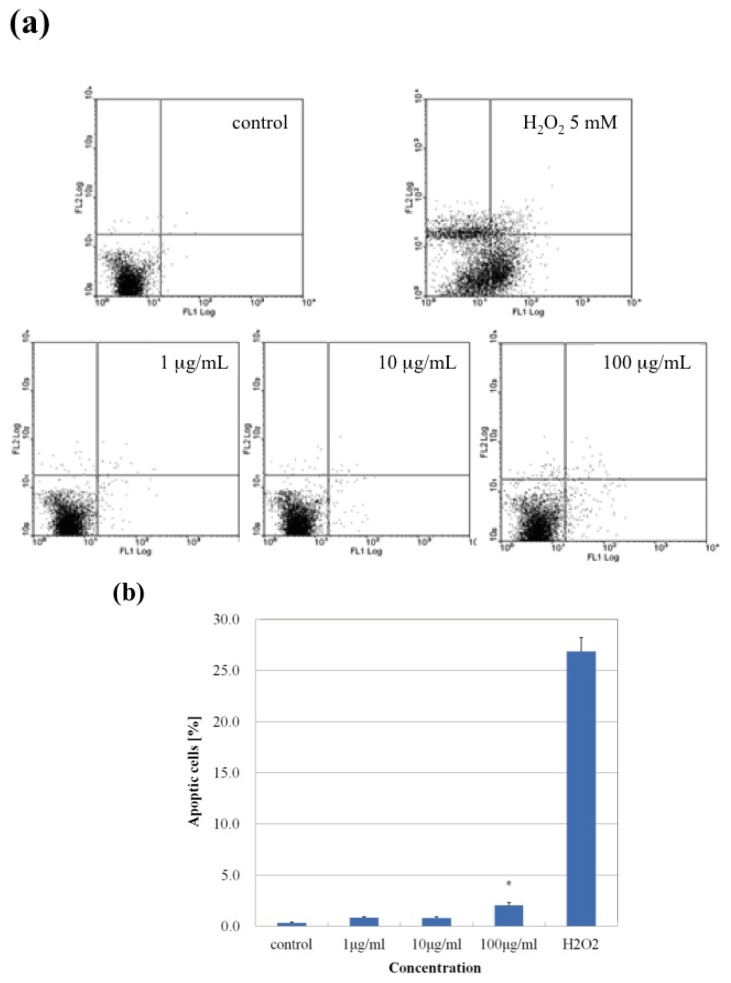
Effect of Fe_3_O_4_ magnetic nanoparticles (MgNPs-Fe_3_O_4_) on apoptosis in A549 cells. A549 cells were treated with 0 (control), 1, 10 or 100 μg/mL MgNPs-Fe_3_O_4_ for 24 h; cells were treated with 5 mM H_2_O_2_ for 24 h as a positive control. Apoptosis of A549 cells treated with MgNPs-Fe_3_O_4_ or H_2_O_2_ was determined by flow cytometry based on propidium iodide/Annexin V staining patterns; (**a**) Representative flow cytometry of one set of triplicate experiments; (**b**) Percentages of apoptotic cells from flow cytometry analysis. Apoptotic cells include early apoptotic cells (AnnexinV+/PI−) and late apoptotic or necrotic cells (AnnexinV+/PI+). Data are presented as the mean ± SD of three independent experiments. * *p* < 0.05 *vs.* control.

Treatment with MgNPs-Fe_3_O_4_ for 24 h did not affect cell viability as assessed by the Alamar Blue assay. However, treatment with 100 μg/mL MgNPs-Fe_3_O_4_ for 72 h caused a significant reduction in cell viability (Figure 3). Significant LDH leakage was detected following treatment with 100 μg/mL MgNPs-Fe_3_O_4_; lower concentrations had no effect (Figure 4). As shown in Figure 5a, treatment with 100 μg/mL MgNPs-Fe_3_O_4_ for 24 h caused a small but significant increase in the percentage Annexin V-staining cells; however, these values were greatly below that caused by H_2_O_2_ (Figure 5b).

### 2.4. Effect of MgNPs-Fe_3_O_4_ on ROS Production, Intracellular Glutathione, and 8-OH-dG Levels in DNA

**Figure 6:**
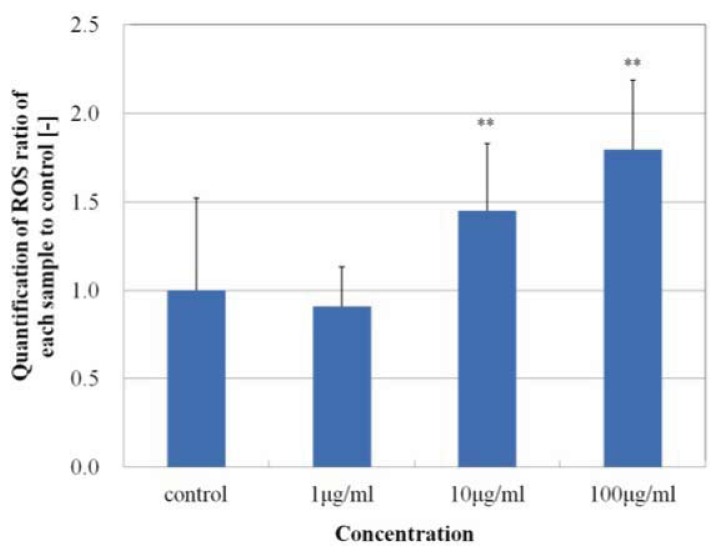
Effect of MgNPs-Fe_3_O_4_ on production of reactive oxygen species (ROS) by A549 cells. A549 cells were treated with doses 0 (control), 1, 10 or 100 μg/mL MgNPs-Fe_3_O_4_ for 24 h. ROS production was determined using the CM-H_2_DCFDA assay. Data are presented as the mean ± SD of three independent experiments. ** *p* < 0.01 *vs.* control.

**Figure 7:**
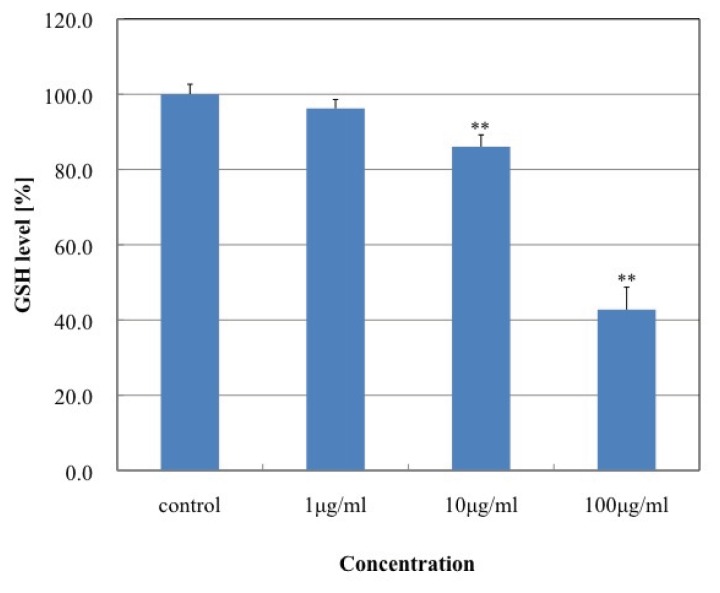
Effect of MgNPs-Fe_3_O_4_ on intracellular glutathione (GSH) levels in A549 cells. A549 cells were treated with 0 (control), 1, 10 or 100 μg/mL MgNPs-Fe_3_O_4_ for 24 h. GSH levels were assessed by luciferin bioluminescence (GSH-Glo Glutathione Assay Kit). Data are presented as the mean ± SD of three independent experiments. Significantly different from the untreated control at ** *p* < 0.01 *vs.* control.

**Figure 8:**
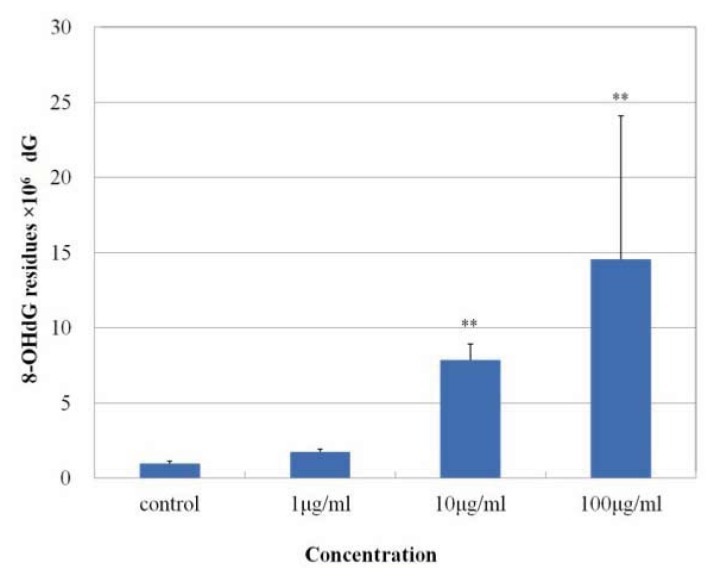
Effect of MgNPs-Fe_3_O_4_ on 8-hydroxy-deoxyguanosine (8-OH-dG) levels in A549 cells. A549 cells were treated with 0 (control), 1, 10 or 100 μg/mL MgNPs-Fe_3_O_4_ for 72 h. DNA was extracted by the sodium iodide method; 8-OH-dG levels were determined using HPLC-ECD. Data are presented as the mean ± SD of three independent experiments. * Significantly different from the untreated control at ** *p* < 0.01 *vs.* control.

As shown in Figure 6, MgNPs-Fe_3_O_4_ caused a dose-dependent increase in ROS production with concentrations of 10 and 100 μg/mL. Figure 7 demonstrates that MgNPs-Fe_3_O_4_ caused a dose-dependent decrease in the GSH level; GSH was reduced by 65% with 100 μg/mL MgNPs-Fe_3_O_4_. The 8-OH-dG levels were increased approximately 8- and 14-fold above control with 10 and 100 μg/mL MgNPs-Fe_3_O_4_, respectively (Figure 8). ROS production by MgNPs-Fe_3_O_4_ is well known to be involved in the cytotoxic response in various cell types. Fe_3_O_4_, a mixture of FeO and Fe_2_O_3_, is unstable and can readily undergo oxidation to yield γ-Fe_2_O_3_ + Fe^2+^ [7,9,16]. The free Fe^2+^ ions can react with hydrogen peroxide and oxygen produced by the mitochondria to produce highly reactive hydroxyl radicals and Fe^3+^ions [17] that can damage DNA, proteins, polysaccharides, and lipids *in vivo*. Similar to our findings, previous studies have shown that Fe_3_O_4_ elicited an increase in oxidative DNA lesions in A549 cells with minimal effect on cell viability [7–9]. Of the non-enzymatic antioxidants, GSH represents the major intracellular redox buffer in all cell types. Abundant in all cell compartments, it constitutes the first line of the cellular defense mechanism against oxidative injury [16]. Previous studies demonstrated that ROS generation following GSH depletion caused mitochondrial damage and up-regulation of pro-apoptosis mediators [2,7,18]. We found MgNPs also significantly reduced the GSH level. However, our data suggest that the shift in balance toward pro-oxidant mechanisms exerts little impact on cell viability.

### 2.5. Expression of the Heme Oxygenase-1 (HO-1) Gene

**Figure 9:**
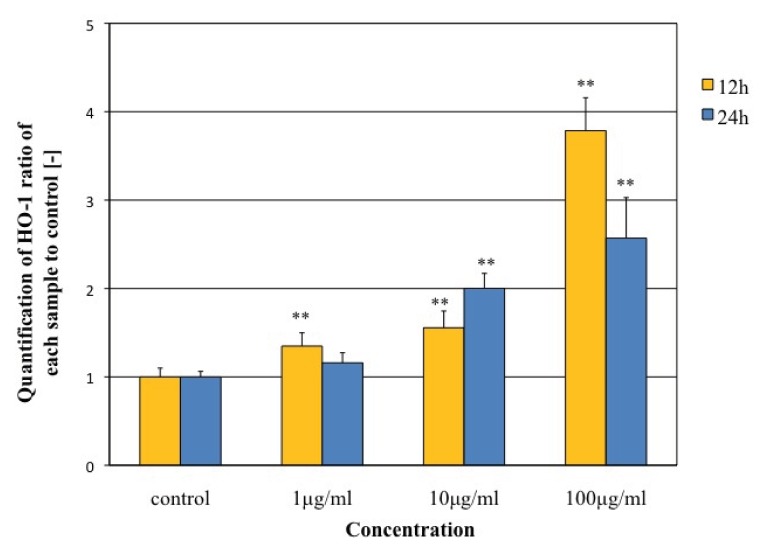
Effect of MgNPs-Fe_3_O_4_ on mRNA expression of the *HO-1* gene in A549 cells. The expression level of the *HO-1* was normalized according to the expression level of β-actin. Data are presented as the mean ± SD of three independent experiments. ** *p* < 0.01 *vs.* control.

As shown in Figure 9, the transcript level of the *HO-1* was induced in a dose-dependent manner after 12 and 24 h of MgNPs-Fe_3_O_4_ exposure, however its transcription level at 100 mg/mL exposure after 24 h was reduced compared to after 12 h. Oxidative stress is caused by an imbalance in the level of ROS and a biological system’s ability to detoxify the reactive intermediates [16]. Cells possess both enzymatic and non-enzymatic mechanisms to counterbalance the cytotoxicity and genotoxicity caused by ROS [16]. In the lungs, the major enzymatic antioxidants are superoxide dismutases (SODs), catalase, and glutathione peroxidase (GSH-Px); others include those examined in this study, HO-1, thioredoxin (TR), and glutaredoxin (GLRX). HO-1 is involved in playing a major role in degradation of heme to biliverdin, but has recognized potent anti-inflammatory and anti-apoptotic effects [17,19]. HO-1 is induced mainly at the transcriptional level by oxidative stress, pro-inflammatory mediators, and some growth factors [18]. HO-1 mRNA expression is known to mediate antioxidant and cytoprotective effects and has been considered useful as a marker for particle-induced oxidative stress. Park *et al.* [20] showed that treatment of a human bronchial epithelial cell line with TiO_2_-MgNPs for four hours caused dose-dependent increases in mRNA expression of HOG-1, glutathione-*S*-transferase, and catalase; mRNA expression level of HO-1 had returned to baseline by 24 h [20]. Napierska *et al.* [21] also showed a marked induction of HO-1 mRNA in the endothelial cell at six hours after treatment of SiO_2_-NPs, but reduction of HO-1 mRNA at 24 h. Our results appear to be same as these two studies.

### 2.6. Effect of MgNPs-Fe_3_O_4_ on CD44^+^ Cell Fraction in A549 Cells

**Figure 10:**
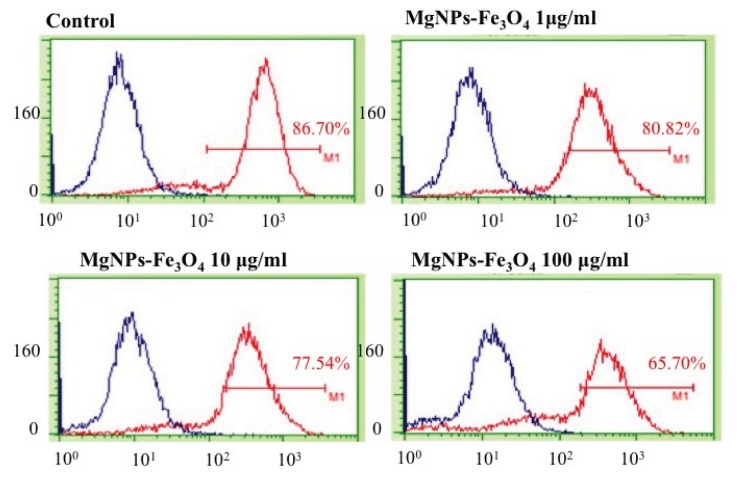
Effect of Fe_3_O_4_ magnetic nanoparticles (MgNPs-Fe_3_O_4_) on CD44^+^ cell fraction in A549 cells. A549 cells were treated with 0 (control), 1, 10 or 100 μg/mL MgNPs-Fe_3_O_4_ for 24 h. Cells were labeled with mouse anti-human CD44 monoclonal antibody; level of CD44^+^ cells was determined by flow cytometry.

MgNPs-Fe_3_O_4_ caused a dose-dependent reduction in the CD44^+^ subpopulation (Figure 10). CD44 is a cell surface glycoprotein that mediates cellular adhesion to the extracellular matrix and is involved in multiple processes, including inflammation, cell migration, signaling, and tumor metastasis [13,22]. CD44 is up-regulated in the damaged epithelium of asthma patients, and is believed to be involved in tissue repair by localizing chemokines and growth factors to the disrupted epithelium [23]. CD44 is also a marker of certain cancer stem cells [24], in which it functions to defend cancer cells against oxidative stress by increasing GSH synthesis [25]. CD44 has also been reported to be involved in the protective effect of hyaluronate on constitutive DNA damage by ROS in A549 cells [26]. Consistent with the previously noted reduction in GSH and increase in 8-OH-dG levels, we found that MgNPs-Fe_3_O_4_ markedly decreased the CD44^+^ cell fraction of A549 cells. Thus, these results highlight another mechanism by which MgNPs-Fe_3_O_4_ impair redox control and damage DNA in A549 cells. Our results also offer the possibility that CD44 may be a marker MgNP-Fe_3_O_4_-induced cytotoxicity; however, further study is warranted.

## 3. Experimental Section

### 3.1. MgNPs-Fe_3_O_4_

MgNPs-Fe_3_O_4_ were obtained from the Toda Kogyo Corporation (Otake, Hiroshima, Japan). As specified by the manufacturer, MgNPs in powder were spherical, with an average particle size of 10 nm, as measured by transmission electron microscopy (TEM), and a surface area of 100–120 m^2^/g. In suspension, the particle size ranged from 60 to 100 nm as measured by dynamic light scattering (DLS) and zeta potential ranged from −30 to −40 mV at pH 10. Bare MgNPs-Fe_3_O_4_ were used in this study.

### 3.2. Preparation of MgNPs-Fe_3_O_4_ in Culture Medium

MgNPs-Fe_3_O_4_ were sterilized by ultraviolet (UV) irradiation and suspended in phosphate-buffered saline (PBS), Ham’s F-12 alone, and Ham’s F-12 medium containing 10% fetal bovine serum (FBS) and 100 U/mL penicillin-streptomycin to yield a concentration of 1, 10 or 100 μg/mL. Suspensions were sonicated at 30 W for 10 min using an Ultrasonic HomogenizerVP-050 (TAITAEC, Koshigaya, Saitama, Japan).

### 3.3. Cell Line

A549 human lung epithelial cells were purchased from American Tissue Type Culture Collection (Manassas, VA, USA). Cells were incubated in Ham’s F-12 Medium with 10% fetal bovine serum (FBS) and 100 U/mL penicillin–streptomycin in 5% CO_2_ at 37 °C. Cells were maintained at a density of 60%–70% confluence in 100 mm dishes, and used in log-phase of growth.

### 3.4. Characterization of MgNP-Fe_3_O_4_ Suspensions in Cell Culture Medium

The average hydrodynamic size and size distribution of MgNPs-Fe_3_O_4_ in cell culture media and their intracellular localization were determined by DLS using a Fiber-Optics Particle Analyzer FPAR-1000 (Otsuka Electronics, Hirakata, Osaka, Japan). MgNPs-Fe_3_O_4_ were suspended in Ham’s F-12 Medium with or without 10% FBS and supplements, or in phosphate-buffered saline (PBS).

### 3.5. Cellular Uptake of MgNPs-Fe_3_O_4_ in A549 Cells

The cellular uptake of MgNPs-Fe_3_O_4_ in A549 cells was analyzed as follows.

#### 3.5.1. Transmission Electron Microscopy (TEM)

A549 cells were fixed with 3% glutaraldehyde in 0.1 M cacodylate buffer (pH 7.3) at 4 °C for 4 h. Samples were post-fixed with 2% osmium tetraoxide at 4 °C for 2 h, dehydrated, and embedded in epoxy resin. Ultrathin sections (80 nm) were then stained with uranyl acetate and lead citrate, and observed by TEM.

#### 3.5.2. Flow Cytometry Assay

A549 cells were treated with 0, 1, 10 or 100 μg/mL MgNPs-Fe_3_O_4_ for 24 h, and then trypsinized and suspended in medium. Cellular uptake of MgNPs-Fe_3_O_4_ was analyzed using flow cytometry (Millipore, Billerica, MA, USA), in which the intensities of forward-scattered (FS) and side-scattered (SS) light are proportional to cell size and intracellular density of MgNPs-Fe_3_O_4_, respectively. A total of 30,000 cells were measured per sample.

### 3.6. AlamarBlue Assay

Cell viability was determined using the alamarBlue assay (Alamar Biosciences, Sacramento, California, USA) according to the manufacturer’s instructions. Briefly, cells (1.0 × 10^4^ cells/well) were incubated with MgNPs-Fe_3_O_4_ (0, 1, 10 or 100 μg/mL) for 72 h at 37 °C. AlamarBlue (10%) was added to each well and incubated for 200 min. Metabolically active cells reduced the dye to a fluorescent form, which was measured using a plate reader (excitation/emission: 570 nm/600 nm; Viento XS, DS Pharma Biomedical, Suita, Osaka, Japan). Cell viability was determined by linear interpolation of the emission from cells treated with 0.1% saponin (0% viability) and untreated cells (100% viability).

### 3.7. Lactate Dehydrogenase (LDH) Release Assay

LDH release assay to assess membrane integrity was performed using LDH-cytotoxicity assay kit (BioVision, CA, USA) according to the manufacturer’s instructions. Cells cultured in 24-well plates (1.5 × 10^4^ cells/well) were treated with 0, 1, 10 or 100 μg/mL MgNPs-Fe_3_O_4_ for 24 h at 37 °C. Plates were then centrifuged at 250× *g* for 5 min. The supernatant of each well was transferred to a fresh, flat bottom 96-well culture plate containing 100 μL reaction mixture, and incubated for 30 min at room temperature. Formazan absorbance—an index of the number of lysed cells—was measured by a microplate reader at 500 nm (Viento XS, DS Pharma Biomedical, Osaka, Japan).

### 3.8. Apoptosis by Flow Cytometry (FCM)

A549 cells (1.0 × 10^6^ cells) were cultured on 100-mm culture dishes, and treated with 0, 1, 10 or 100 μg/mL MgNPs-Fe_3_O_4_ for 24 h at 37 °C. Cells were harvested, washed gently with PBS, collected by centrifugation, and then stained using an Annexin V-FITC Kit (Beckman Coulter, Marseille, France) following the manufacturer’s instructions. Cells were stained with Annexin V and propidium iodide (PI, Sigma-Aldrich, St. Louis, MO, USA), and analyzed by flow cytometry (Becton Dickinson, Franklin Lakes, NJ, USA) within 1 h of staining using the FL1 (FITC) and FL3 (PI) lines.

### 3.9. Measurement of Intracellular Reactive Oxygen Species (ROS)

ROS were measured using the CM-H_2_DCFDA assay (Invitrogen, Carlsbad, CA, USA) according to the manufacturer’s instructions. Cells (1.0 × 10^4^ cells/well) in 24 well-plates were treated with 0, 1, 10 or 100 μg/mL MGNPs-Fe_3_O_4_ for 24 h at 37 °C. A fresh stock solution of 5 mM CM-H_2_DCFDA was prepared in DMSO and diluted to a final concentration of 1 μM in PBS. Cells were washed with PBS, followed by incubation with 50 μL of working solution of the fluorochrome marker CM-H_2_ DCFDA for 30 min. Fluorescent images were obtained using an IX2N-FL-1 microscope (Olympus, Tokyo, Japan), and analyzed using imaging soft (Photoshop Elements 8, Adobe systems, Tokyo, Japan). The data were expressed as percentage of the unexposed control.

### 3.10. Intracellular Reduced Glutathione (GSH) Assay

Intracellular GSH level was determined using a GSH-Glo Glutathione assay kit (Promega, Madison, WI, USA) according to the manufacturer’s instructions. Briefly, cells were seeded in 96-well plates and treated with 0, 1, 10 or 100 μg/mL MgNPs-Fe_3_O_4_ for 24 h at 37 °C. The cells were washed with DPBS, and the GSH-Glo reagent was added to each well for 30 min at room temperature to allow the cells to convert a luciferin derivative into luciferin. Reconstituted luciferin detection reagent was then added to each well for 15 min, and the luminescent signal was measured with a Glomax multi detection system (Promega, Madison, WI, USA).

### 3.11. Analysis of 8-Hydroxy-2′-Deoxyguanosine (8-OH-dG) in DNA

A549 cells were incubated with 0, 1, 10 or 100 μg/mL MGNPs-Fe_3_O_4_ for 72 h at 37 °C (5% CO_2_). The nuclear DNA was isolated by the sodium iodide method. The 8-OH-dG levels were analyzed by HPLC-ECD methods as previously described [27]. The amount of 8-OH-dG in the DNA was determined by comparison to authentic standards, and expressed as the number of 8-OH-dG per 10^6^ deoxyguanosine (dG) residues.

### 3.12. Oxidative Stress-Related Gene Expression Analysis

A549 cells were treated with 0, 1, 10, or 100 μg/mL MgNPs-Fe_3_O_4_ for 24 h at 37 °C. Total RNA was isolated using ISOGEN (Nippon Gene, Tokyo, Japan), and cDNA was produced using a mixture containing Superscript RNase H Reverse Transcriptase (Invitrogen, Carlsbad, CA, USA), oligo dT primer, and 2.5 mmol/L dNTP. Quantitative real-time PCR was conducted using the LINE GENE real-time PCR detection system (BioFlux, Tokyo, Japan) with the SYBR Premix Ex Taq Perfect Real Time Kit (Takara Bio. Inc., Otsu, Japan). The PCR reaction consisted of initial thermal activation at 95 °C for 10 s and 40 cycles. Each cycle was as follows: 95 °C for 5 s; 60 °C for 26 s. PCR producs were verified by analysis of heat-dissociation curves and amplification plots. Quantitative values were acquired from linear regression of the PCR standard curve. The primer sequences of the amplified genes are as follows [28,29]; *Heme oxygenase-1*, forward 5′-GGTGATAGAAGAGGCCAAGAC-3′ and reverse 5′-GCAGAATCTTGCACTTTGTTG-3′, β*-actin*, forward 5′-GGATGCAGAAGG AGATCACTG-3′ and reverse 5′-CGATCCACACGGAGTACTTG-3′.

### 3.13. Immunostaining and Flow Cytometric Analysis for CD44^+^ Cell Fraction

A549 cells were treated with 0, 1, 10 or 100 μg/mL MgNPs-Fe_3_O_4_ for 24 h at 37 °C. Cells were then labeled in a PBS solution with a mouse anti-human CD44 monoclonal antibody conjugated with fluorescein isothiocyanate (clone SFF-2, Millipore, Billerica, MA, USA) for 1 h at room temperature. A mouse IgG immunoglobulin and dye conjugate IgG was used as control for non-specific binding. Flow cytometric analysis was performed with a Guava-EasyCyte*HT using Guava Express Pro software (Millipore, Billerica, MA, USA) gating for CD44^+^ cells. A minimum of 10,000 cells was measured per sample.

### 3.14. Statistical Analysis

Data are presented as the mean ± standard deviation (SD). Differences between treated and untreated control cells were determined using one-way ANOVA followed by Dunnett’s test. Differences were considered statistically significant at *p* < 0.05.

## 4. Conclusions

MgNPs-Fe_3_O_4_ up to a concentration of 100 μg/mL exerted minimal effect on viability of A549 cells, despite causing a significant reduction in antioxidant capacity and an increase in oxidative damage to DNA. Increased expression of an oxidative stress-related gene was not sufficient to prevent the decrease in GSH content. The decrease in the CD44^+^ cell fraction was consistent with the observed drop in GSH concentration and increase in 8-OH-dG level.
